# Constituent of extracellular polymeric substances (EPS) produced by a range of soil bacteria and fungi

**DOI:** 10.1186/s12866-025-04034-z

**Published:** 2025-05-15

**Authors:** Rebeca Leme Oliva, Umesh B. Khadka, Tessa Camenzind, Jens Dyckmans, Rainer Georg Joergensen

**Affiliations:** 1 Soil Biology and Plant Nutrition, University of Kassel, Nordbahnhofstr. 1a, Witzenhausen, 37213 Germany; 2 Institute of Biology, Freie Universität Berlin, Altensteinstr. 6, Berlin, 14195 Germany; 3 Centre for Stable Isotope Research and Analysis, University of Göttingen, Büsgenweg 2, Göttingen, 37077 Germany

**Keywords:** Biofilm, EPS, Amino sugars, Total proteins, Total carbohydrates, Extracellular dsDNA

## Abstract

Extracellular polymeric substances (EPS) produced by soil bacteria and fungi are crucial for microbial growth and provide many functions for the soil and its microbes. EPS composition may depend on microbial community composition and the soil physical and chemical environment, nevertheless, not much is known about the EPS constituents’ specific roles nor how they interact to alter biofilm’s functions. We hypothesized that EPS production would be enhanced with the presence of a surface and with a more labile carbon source. Also, that even though carbohydrates and proteins are the main constituents of EPS, we could still find quantifiable amounts of mannosamine and galactosamine (two amino sugars previously shown to be part of microbial biofilms). Ten soil bacterial and ten soil fungal species were cultured with glycerol or starch and with or without a quartz matrix. After a 4-day cultivation, EPS were extracted, and seven constituents were determined: carbohydrates, DNA, proteins, muramic acid, mannosamine, galactosamine, and glucosamine. We found EPS composition was strongly modified by microbial type, whereas differences in EPS production were driven mostly by environmental conditions. The EPS-carbohydrate/protein ratio was higher for cultures grown in starch media than in glycerol and increased in the presence of quartz. EPS-carbohydrate concentration reflected environmental changes of substrate quality and surface presence. Contrastingly, changes in the other EPS constituent composition are likely due to intrinsic microbial characteristics. Our findings open the pathway to study microbial biofilms in more complex environments (such as soils) and shed light to the importance of extracellular structures to microbial processes.

**Supplementary Information:**

The online version contains supplementary material available at 10.1186/s12866-025-04034-z.

## Background

Most microorganisms live within a biofilm matrix of extracellular polymeric substances (EPS) [1–3]. EPS form a scaffolding structure, allowing cells to attach to surfaces and maintain a cohesive unit [4]. Due to the interest in studying biofilms in biotechnology [5], medical [6], aquatic [7] and soil [8, 9] sciences, much knowledge has been gained regarding EPS production and composition in recent years. Nevertheless, mostly because of methodological constraints, it has been hard to determine which environmental factors determine EPS production and compositions, and how specific EPS constituents affect their functions [10]. Biofilms perform different functions beyond being a structure for microorganisms to grow within. They also help with nutrient and water retention [2, 11–13] and protection of extracellular enzymes [11]. When biofilms are formed in soils, they additionally offer a series of benefits for the soil ecosystem, like increasing and strengthening aggregate stability [9, 14, 15] and improving the resistance of soil against drought and salinity [16–19].

EPS are mainly composed of carbohydrates and proteins, but other substances such as lipids, amino sugars, and DNA have also been found within the matrix [6, 20]. The composition of biofilms depends on the type of microorganism, substrate availability, and environmental conditions [21]. Biofilm composition can, in turn, directly determine the physical and functional properties of biofilms [6, 10]. For example, EPS-carbohydrates and EPS-proteins have been repeatedly reported to create the architecture of biofilms, to serve as nutrient source, and to aid cell-surface attachment [10, 21], even though the specific mechanisms behind these properties have not yet been clarified. Further, the extracellular DNA present in biofilms is thought to promote horizontal gene transfer between cells, contributing to evolutionary fitness and overall resistance of microbial communities against environmental disturbances [21, 22]. At the same time, little to no knowledge is available on EPS-amino sugars and how they interact with other constituents to reflect changes in biofilm properties, despite their recognized importance to the microbial residual fraction [20, 23, 24].

Amino sugars (AS), i.e., muramic acid (MurN), mannosamine (ManN), galactosamine (GalN), and glucosamine (GlcN), are all important markers of microbial residues, a fraction which encompasses all non-living microbial products, such as EPS and necromass, i.e., dead cell remains [25]. Furthermore, AS are already remarked as important microbial markers in soil studies as they contribute 5–12% to total N [26] and 2–5% to soil organic carbon [27]. GlcN and MurN are known cell-wall components and widely used as indicators of necromass [25], whereas the GalN and ManN contents found in soils lack a similar clear meaning. This will change as recently Oliva et al. [20] showed that GalN and ManN are exclusively derived from microbial EPS. However, this same study presented limitations as results were obtained based on just two cultured bacterial and two fungal species [20]. In addition, they found also significant concentrations of GlcN and MurN with unknown functions in the EPS. Studying the role of all four amino sugars as EPS constituents might be crucial to understand the importance of biofilms in the long-term C storage in complex environments such as soils as well as how microbes stabilize and recycle necromass within the biofilm structure.

In order to better understand the contribution of environmental factors to EPS production and composition (especially regarding the interaction of EPS-AS with other components), we extracted EPS from ten bacterial and ten fungal species grown in either glycerol or starch media with or without a quartz matrix. We have further analyzed the composition of the extracted EPS, quantifying a total of seven compounds: total carbohydrates, total proteins, DNA and the AS: MurN, ManN, GalN, and GlcN. With this controlled design we tested the following hypotheses: (1) more EPS are produced in cultures grown in a more labile carbon source, showing that microbial EPS formation and composition depend on the substrate quality. (2) The production of microbial EPS constituents is increased in the presence of a mineral surface for attachment, supporting the idea that EPS production might be crucial in environments such as soils. (3) MurN in EPS indicates the presence of bacterial necromass, whereas GlcN could be also part of EPS such as GalN and ManN. Investigating the effects of substrates and environmental conditions on microbial biofilm production is an important step to reveal how biofilms work in more complex environments, such as soils. Our study should therefore help disentangle the role of microbial EPS in soil processes.

## Methods

### Microbial strains

In this study, ten bacterial and ten fungal species were cultured under different conditions to induce EPS production (S1). The species were chosen following two criteria: first, they should belong to biosafety level 1; second, they should either be commonly found in soils or have been isolated from soils. The list containing detailed information on the selected microbial species can be found in the Supplementary Material (S2). Nine bacterial strains were acquired from the German Collection of Microorganisms and Cell Cultures (DSMZ) and only *Escherichia coli* (MG1655) was obtained in cooperation with the Freie Universität Berlin. All ten fungal strains were isolated from an agricultural soil, i.e., the unfertilized plots of the long-term field experiment in Thyrow. They were identified with the use of molecular techniques as described in Supplementary-Material S3 (for more details see also ref. 20). Some of the species used in the present study were also cultivated for another experiment carried out by the authors (ref. 20). Nevertheless, those cultures were not identical to those used in the current study, but rather, each species was inoculated again, following the method description given in the following sections.

### Microbial growth conditions

Four replicates of each microbial species were individually cultured in starch or glycerol media as described by Oliva et al. [20], using 500 ml shake flasks either with or without a matrix of SOM-free sterile quartz (SiO_2_, 0.4–0.8 mm – Carl Roth). Microbial strains were kept in agar plates prior to the experiment. On the day of incubation, four inocula were taken from each species’ agar plate and with the help of an inoculation hoop, transferred to the 500 ml shake flasks used in this study. Each flask was subsequently filled with 50 ml of culture medium and 140 g of quartz (when necessary). The ratio of liquid medium to quartz was determined with the aim to provide medium for microbial growth without nutrient restriction, while still physically forcing them to grow within the quartz matrix. Meaning the quartz matrix was not just at the bottom of the flasks as the microorganisms were left to float in liquid media above it, but rather that there was enough liquid medium to force microbial strains to physically grow within the quartz matrix. Flasks were incubated at 30 °C and shaken (100 rev min^−1^) for 4 days until EPS extraction ensuring adequate homogeneity and aeration during microbial growth.

### EPS extraction and analysis of constituents

After a four-day incubation, the EPS fraction was extracted from cell cultures using the method proposed by Frølund et al. [28] and further explained at Oliva et al. [20]. The presence of biofilms was not confirmed by microscopy before extraction, as the focus of our study was not to distinguish between the morphology of the biofilm and its components, such as proteins, carbohydrates, dead cell remains, and bacterial capsules, but the interest was rather the composition of the extracellular environment as a whole. For this reason, the data were also not normalized to other microbial-specific units such CFU counts or biomass. We started by collecting 10 ml aliquots from the cell cultures (both with and without quartz) with the help of a graduated standing cylinder and followed the protocol adding the recommended amount of cation exchange resin (CER, Amberlite® HPR1100, Sigma-Aldrich) determined for cultures of *Pseudomonas putida* [28]. After extraction, the resulting EPS was stored in −20 °C until further analysis.

Altogether, four chemical compounds were analyzed in the extracted EPS: (1) total carbohydrates (EPS-carbohydrates), (2) total proteins (EPS-proteins), (3) DNA (EPS-DNA), and (4) amino sugars (EPS-amino sugars). Total carbohydrate was determined as described by Bublitz et al. [29]. 2 ml of a 0.75 M H_2_SO_4_ solution was added to EPS aliquots in a 1:1 ratio (v/v) and hydrolyzed in an autoclave for 10 min at 100 °C. Afterward, the hydrolysates were diluted with phosphate saline buffer (PBS – same buffer used to store EPS extracts) until reaching the maximum limit of detection, making the neutralization step proposed by Bublitz et al. [29] unnecessary. Then, total carbohydrates were estimated with the bicinchoninic acid (BCA) microplate assay, measuring absorbance at 562 nm [29]. Additionally, total protein content was estimated using the Lowry assay microplate method, as described by Redmile-Gordon et al. [30]. For protein determination, EPS extracts were incubated with a copper sulphate solution (CuSO_4_ × 5 H_2_O), containing the Folin-Ciocalteu-reagent, before the absorbance was recorded at 750 nm.

DNA was purified from the EPS extracts initially by adding 0.525 ml of a phenol:chloroform:isoamyl alcohol solution (24:25:1 v/v/v) to 0.5 ml of EPS extract [31]. The mixture was gently stirred by inversion for 10 min and subsequently centrifuged for 15 min at 12,000 rpm [31]. After carefully extracting the upper liquid phase (without disturbing the formed bilayer) and transferring it to a new tube, 0.07 ml of a 3 M sodium acetate solution and 0.7 ml of isopropanol were added and tubes were centrifuged for 25 min at 12,000 rpm to precipitate DNA [32]. The supernatant was discarded from the tubes and washed with 0.7 ml of ethanol, followed by a last centrifugation step for 25 min at 12,000 rpm*)* [32]. After ethanol was discarded and the pellet allowed to dry, purified DNA samples were resuspended in 0.05 ml of Tris–EDTA buffer and stored at −20 °C until further analysis. Lastly, the concentration of DNA was determined using the Pico488 dsDNA quantification reagent kit (Lumiprobe Life Science Solutions, Germany) by fluorescence detection at 525 nm.

To determine amino sugar content, a 2 ml aliquot of the EPS samples was hydrolyzed with 2 ml of a 6 M HCl solution in an autoclave for 10 min at 100 °C, as described by Oliva et al. [20]. Subsequently, the concentrations of muramic acid (MurN), mannosamine (ManN), glucosamine (GlcN), and galactosamine (GalN) was determined by high performance liquid chromatography (HPLC) according to Appuhn et al. [33] as described by Indorf et al. [34]. Sample derivatization was performed with ortho-phthaldialdehyde in a Dionex (Germering, Germany) HPLC Ultimate WPS-3000 TSL analytical autosampler with in-line split-loop injection and thermostat, coupled to an Ultimate 3000 pump and an Ultimate 3000 fluorescence detector set at 330 nm excitation and 445 nm emission wavelengths.

### Statistical analysis

Our dataset was analyzed using the R Statistical Software (v4.4.2, [35]). The dataset included seven EPS constituents (proteins, carbohydrates, DNA, MurN, ManN, GalN, and GlcN) and four factors: species (20 levels), microbial type (2 levels: bacteria and fungi), matrix (2 levels: with and without quartz) and substrate (2 levels: glycerol and starch). The quantified amounts of different EPS components were all converted to µg ml^−1^ of microbial suspension (except DNA, converted to ng ml^−1^ of microbial suspension), and therefore express the gross value of each component produced in 50 ml of incubation medium after 4 days. Using the outliers R package (v0.15, [36]), a Dixon test was carried out to detect and to remove significant (*P* < 0.05) outliers from the dataset. Afterwards, all variables were standardized and normalized using the ordered quantile (ORQ) normalization with the BestNormalize R package (v1.9.0, [37]). Following data normalization, we carried out a mixed-effect linear model analysis (with the lme4 R package—v1.1.34, [38]), using species as a random factor, whereas microbial type, matrix, and substrate were used as fixed factors. Results with *P* < 0.05 were considered significant effects. We chose to use species as a random factor due to the following reasons: (1) The variation in EPS production was high among the different species. (2) The patterns of EPS production within functional groups, e.g., bacteria vs. fungi, were more interesting and in line with our research objectives. Nevertheless, a detailed table with results per species can be found in Supplementary-Material S4. All data (replicates were not averaged beforehand) were plotted as box plots, using the SigmaPlot 13.0 (Systat, San José, USA). The EPS-carbohydrate/EPS-protein ratio was also calculated, simply by dividing quantified carbohydrate and protein amounts in each sample. This ratio has been linked to biofilm viscosity [39] and is thought to provide meaningful information on the origin of EPS [40].

Lastly, we performed a principal component analysis (PCA), using the stats package (v4.4.2, [35]), aiming to reduce the data complexity and to visually explore the correlations among different components. In total five PCA graphs were created, three for the whole dataset (grouped either by type, matrix or substrate), one for the bacterial and one for the fungal dataset.

## Results

### Substrate, matrix, and microbial type effects on EPS constituents

**Fig. 1:**
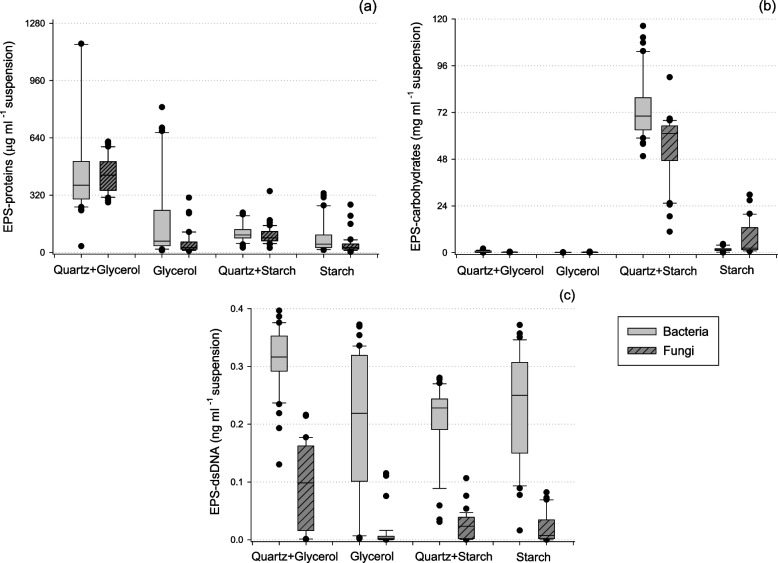
Quantified amounts of (**a**) proteins, **b** carbohydrates and (**c**) DNA in the extracted EPS fraction from bacterial and fungal cultures grown under contrasting substrate and matrix treatments. Different color boxplots represent bacterial and fungal EPS production. Replicates have not been averaged to produce this graph

EPS composition was significantly affected by carbon sources, microbial group, and the presence of a quartz matrix. EPS-protein was higher in cultures grown in a more labile carbon source and in the presence of a surface (Quartz + glycerol treatment—Fig. 1a, Table 1). Bacterial and fungal cultures grown in glycerol medium showed increased EPS-protein production compared with cultures grown in starch medium (*P* < 0.01). Also, cultures grown in the presence of a quartz matrix produced more EPS-protein compared with cultures grown in liquid medium (*P* < 0.01). Further, bacterial species produced on average more EPS-protein than fungal species across all treatments (Table 1). EPS-carbohydrate production was dramatically higher for both bacterial and fungal species in the starch + quartz treatment than in any other treatment (Fig. 1b, Table 1). Additionally, starch led to increased EPS-carbohydrate production in both bacterial and fungal cultures. The EPS-carbohydrate/protein ratio was increased in starch medium (Table 2). The EPS-carbohydrate/protein ratio also increased in the presence of the quartz matrix in most cases, independent of the culture substrate.

**Table 1:** Quantified amounts of EPS components in fungal and bacterial cultures grown under different substrate and matrix treatments. Amounts are expressed in µg ml^−1^ of cell culture after 4 days of incubation, with the exception of DNA, expressed in ng ml^−1^ of cell culture. Coefficient of variation (CV%) was estimated for each 4 replicates (*n* = 4)

		Proteins	Carbohydrates	MurN	ManN	GalN	GlcN	DNA
		Average amount quantified in the EPS fraction (µg ml^−1^ of cell culture)						(ng ml^−1^ of cell culture)
Bacteria	Glycerol	167	61	2.2	11.3	5.1	10.6	0.21
	Glycerol + quartz	510	512	14.0	19.9	5.0	23.6	0.31
	Starch	89	1606	1.5	9.4	4.2	8.2	0.23
	Starch + quartz	108	74,418	7.0	10.1	3.8	11.6	0.21
Fungi	Glycerol	57	2172	ND	5.4	2.1	3.2	0.01
	Glycerol + quartz	438	198	ND	5.3	2.4	6.2	0.09
	Starch	42	6727	ND	5.6	2.2	5.0	0.02
	Starch + quartz	96	54,958	ND	5.8	2.5	7.3	0.02
Probability values								
Matrix		<.01	<.01	<.01	<.01	<.01	<.01	<.01
Substrate		<.01	<.01	<.01	<.01	.03	NS	<.01
Microbial type		.03	NS	–	<.01	<.01	<.01	<.01
Matrix x Substrate		<.01	<.01	.01	NS	NS	<.01	<.01
Matrix x Type		<.01	<.01	–	.04	.01	<.01	<.01
Substrate x Type		NS	<.01	–	<.01	<.01	<.01	.02
CV (%)		22	14	14	4	7	12	32

**Table 2:** Calculated ratios among different EPS components. The carbohydrate/protein ratio was calculated using absolute quantified amounts (in µg ml-1 of cell culture) whereas GlcN/MurN and GlcN/GalN are molar ratios, determined with the use of each amino sugar’s molecular weight

		Carbohydrate/Protein	GlcN/MurN	GlcN/GalN
Bacteria	Glycerol	1	6	2
	Glycerol + quartz	1	2	5
	Starch	53	8	2
	Starch + quartz	856	2	3
Fungi	Glycerol	9	-	1
	Glycerol + quartz	0.5	-	3
	Starch	233	-	2
	Starch + quartz	734	-	3

Bacterial cultures displayed significantly higher EPS-DNA concentration compared with fungal cultures (*P* < 0.01, Fig. 1c, Table 1). Further, the presence of quartz caused increased EPS-DNA production in bacterial cultures only in glycerol medium (Fig. 1c); whereas for cultures grown in starch, the quartz matrix had no significant effect in EPS-DNA production.

**Fig. 2:**
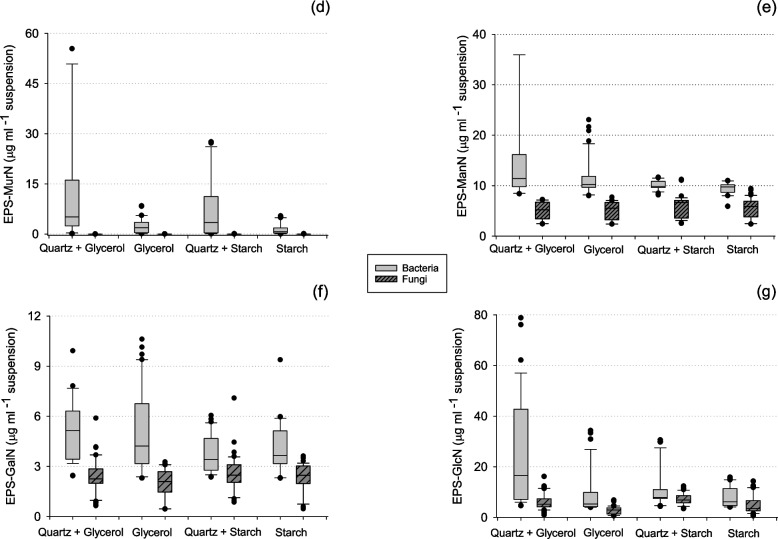
Quantified amounts of (**d**) muramic acid (MurN), **e** mannosamine (ManN), **f** galactosamine (GalN) and (**g**) glucosamine (GlcN) in the extracted EPS fraction from bacterial and fungal cultures grown under contrasting substrate and matrix treatments. Different color boxplots represent bacterial and fungal EPS production. Replicates have not been averaged to produce this graph

Additionally, EPS-amino sugar production also changed significantly with the different factors. Similar to EPS-protein, higher EPS-MurN concentrations were found in bacterial cultures grown in a glycerol medium (*P* < 0.01) and in the presence of the quartz matrix (*P* < 0.01) (Fig. 2d, Table 1). Fungal cultures did not contain detectable concentrations of EPS-MurN. Higher EPS-ManN and EPS-GalN concentrations were found in bacterial than in fungal cultures (*P* < 0.01, Fig. 2e and f, Table 1). However, there was no strong effect of substrate or matrix on EPS-GalN and EPS-ManN concentrations. Bacterial cultures contained, again, significantly higher EPS-GlcN concentrations than fungal cultures (*P* < 0.01, Fig. 2g, Table 1). The quartz matrix generally induced higher EPS-GlcN, regardless of substrate.

Lastly, the molar EPS-GlcN/MurN ratios were constant at 2:1 for bacterial cultures grown in the presence of quartz, but higher for cultures grown in liquid media, namely, 6:1 in glycerol and 8:1 in starch media (Table 2). The molar EPS-GlcN/GalN ratios were higher for bacterial and fungal cultures grown in the presence of quartz than for cultures grown in liquid media (Table 2). The mean EPS-GlcN/GalN ratio was 2 for cultures grown in liquid media, except for fungi grown in glycerol media, where it was 1. For cultures grown with quartz matrix, the EPS-GlcN/GalN ratios were 3, except for bacteria grown in glycerol + quartz media, where it was 5.

### Relationships between EPS constituents

**Fig. 3:**
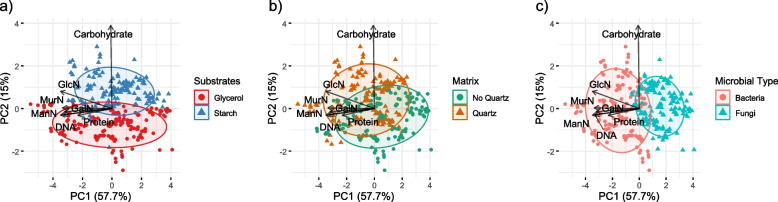
Principal Component Analysis (PCA) of the whole data set. While arrows spatially represent the variation of the different EPS constituents; ellipses represent the studied factors with a confidence interval of 95%: **a** substrate (either glycerol or starch), **b** matrix (either with or without quartz) and (**c**) microbial type (either bacteria or fungi)

In the PCA of the whole dataset (Fig. 3), the two principal components were able to explain 72.7% of the data variation between the 7 constituents (57.7% by PC1 and 15.0% by PC2, respectively). Variability captured by PC1 mainly represented strong differences among the microbial groups (Fig. 3c), driven by amounts of protein, DNA, ManN, GalN, and MurN in EPS. GlcN presented only moderate importance in this axis and that of carbohydrates was completely absent in PC1. In contrast, EPS carbohydrate concentration caused variability among treatments primarily represented by PC2 (Fig. 3a, b). The treatment starch + quartz positioned highest along the axis of PC2, whereas the sole glycerol treatment displayed the lowest eigenvalues.

**Fig. 4:**
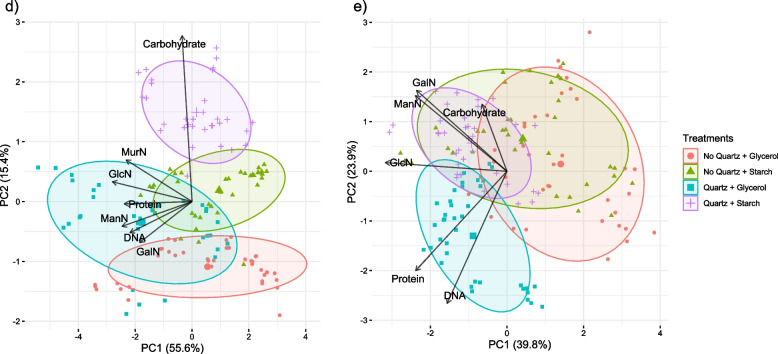
Principal Component Analysis (PCA) of (**d**) bacterial and (**e**) fungal EPS composition. While arrows spatially represent the variation of the different EPS constituents; ellipses represent the studied factors with a confidence interval of 95%: substrate (either glycerol or starch) and matrix (either with or without quartz)

In the PCA of the sole bacterial dataset (Fig. 4d), the two principal components were able to explain 71.0% of the data variation between the 7 constituents (55.6% by PC1 and 15.4% by PC2, respectively). The relationships were similar to those in the PCA of the whole dataset. PC2 was heavily dependent on EPS-carbohydrates while the other variables play little to no significant role. In contrast, PC1 displays higher importance for the other five constituents. Unlike the PCA for the whole dataset, variables were less strongly related in bacterial cultures as indicated by more spaced arrows.

In the PCA of the sole fungal dataset (Fig. 4e), the pattern of relationships among EPS constituents was markedly different. The two principal components were able to explain 63.7% of data variation between the 7 constituents (39.8% by PC1 and 23.9% by PC2, respectively). The main difference was the lack of relationships between EPS-GlcN and the other constituents, whereas EPS-carbohydrates were positively related to EPS-GalN and EPS-ManN. In addition, these two amino sugars were negatively related to EPS-protein and EPS-DNA.

## Discussion

### EPS-carbohydrates and EPS-protein

Our results indicate that particularly the EPS-carbohydrate/protein ratio could be a powerful indicator of EPS functions. The EPS-carbohydrate/protein ratios were generally higher in EPS extracted from cultures grown in starch compared with those grown in glycerol media. In aquatic environments, higher EPS-carbohydrate/protein ratios have been associated with less viscous and more hydrophobic biofilms [39]. In turn, biofilms composed of more hydrophobic molecules can present resistance against water, solvents, and biocides [41, 42] due to an enhanced adsorptive capacity of organic compounds [11]. More complex substrates (more recalcitrant, which require more energy to decompose) can trigger microbial communities to produce biofilms with higher molecular hydrophobicity, increasing their water-resistance, adsorption, and particle aggregation capabilities. Contrastingly, in cultures grown in glycerol medium, lower EPS-carbohydrate/protein ratios can lead to more viscous and hydrophilic biofilms [39]. The presence of hydrophilic polysaccharides and proteins in microbial biofilms is believed to promote biofilm water retention, resulting in communities with a higher tolerance to drought in dry environments [11]. Nevertheless, this view currently lacks experimental evidence as our results on their own do not provide sufficient support.

Additionally, the surface presence shifted EPS-carbohydrate/protein ratios. In most cases, the quartz matrix led to increased EPS-carbohydrate/protein ratios (Table 1), indicating that carbohydrates might be driving biofilm adhesion to solid surfaces. In accordance with our results, in *Bacillus subtilis* and *Pseudomonas aeruginosa* cultures, higher polysaccharide presence in the EPS was correlated with stronger EPS adhesion capacity and biofilm growth [43]. Nevertheless, a study conducted with *Lactobacillus rhamnosus* observed that cell adherence to biofilms was dependent on specific interactions between surface proteins and fatty acids present in the cell envelope, rather than in the extracellular environment [44]. Despite this conflict, our results point to a potential pathway for understanding microbial biofilm adhesion in more complex environments, like soils. Even though quartz lacks reactivity, our results open the path for future studies to assess EPS composition in more complex settings.

Lastly, exponentially more EPS-carbohydrates were quantified in cultures grown in starch + quartz compared with all other treatments, which could be due to methodological constraints. The CER extraction is the most widely accepted method for EPS extraction across a variety of sample types [15, 28, 45, 46]. The CER extractant can be easily removed via centrifugation and there is no addition of chemical compounds that could interfere with EPS composition. However, the method lacks abrasiveness and might only be able to extract loosely bound EPS [46]. This creates doubts whether the measured carbohydrate concentrations accurately reflect only the biofilm structure or whether non-metabolized starch could have been co-extracted. Even though the centrifugation steps should separate culture medium from cells and EPS during the extraction protocol, we only quantified total carbohydrates [29], making a separation between unmetabolized starch and other polysaccharides impossible. Nonetheless, the trends and major results discussed so far are still valid and point to meaningful advances towards better understanding the composition and function of microbial EPS.

### EPS-DNA

Extracellular DNA is commonly found in EPS [1, 3, 11] and thought to originate either from active microbial secretion or death [47–49]. In our study, the presence of quartz significantly increased EPS-DNA concentrations (*P* < 0.01). This suggests that the presence of a surface not only results in higher biomass turnover into necromass, but also that EPS-DNA may have a role in the process of cell surface-adherence. Moreover, the presence of more labile carbon sources as EPS could trigger the release of dsDNA, which could affect microbial cell adherence, a hypothesis explored below.

Similar results have been widely found for bacterial cultures such as *P. aeruginosa* [50], *Streptococcus* sp. [51] and *B. cereus* [47]. Even though the release mechanism of dsDNA into the extracellular environment is still unclear, EPS-DNA seems to be important for bacterial cell adhesion, whereas the EPS-DNA of fungal cultures seems to indicate biofilm formation and hyphal transformation [52, 53]. For instance, in *Candida albicans* cultures, the addition of DNA to biofilms induced hyphal formation and biofilm production [52]. In *Aspergillus fumigatus* cultures, EPS-DNA induced biofilm production and triggered spore adherence to surfaces, which resulted in the growth of hyphal networks within the biofilm matrix after germination [53]. Nevertheless, in our study, it is unlikely that fungal species had enough time for sporulation and for spore germination during the four-day incubation period [54, 55]. This suggests that the fungal EPS-DNA mainly originated from fungal necromass.

Another important finding was that bacterial EPS presented significantly higher amounts of EPS-DNA when compared to fungal EPS (Table 1), regardless of treatment. This was partly expected because bacteria contain markedly more DNA than fungi [56].

### EPS-amino sugars

EPS-ManN and EPS-GalN concentrations were relatively constant within bacteria and fungi, suggesting that they might be attached and regulated by the physio-chemical properties of the cell walls and that their secretion did not depend on environmental conditions. Bacteria produced more EPS-ManN and EPS-GalN than fungi across all treatments, similarly to EPS-DNA, indicating either microbial necromass, i.e., dead cells, or quorum sensing initiatives within the microbial biofilm. ManN is a known constituent of sialic acids in bacteria but also eukaryotes, often found as part of glycoproteins or glycolipids attached to the end of sugar chains on cell surfaces [57, 58]. GalN, on the other hand, had its origin unknown until very recently, when it was shown to be an integral part of EPS [20] but also of teichoic acids, densely attached to the cell surface of Gram-positive bacteria [25]. Our results point to the need to further understand the role of ManN and GalN in the extracellular environment of soil microorganisms.

EPS-MurN was produced in higher concentrations in bacteria grown with quartz. As MurN only occurs in the peptidoglycan layer of bacterial cell walls, we suggest that EPS-MurN is mainly an indicator for bacterial necromass, due to a quartz-induced increased abrasion during extraction. We also observed similar patterns for EPS-GlcN, which are mainly cell-wall constituents of bacterial murein and fungal chitin [25]. This suggests that EPS-GlcN also indicates microbial necromass within the biofilm matrix. However, in bacterial cell-walls GlcN and MurN should occur at a 1:1 molar ratio [59], suggesting that the excess GlcN is derived from microbial EPS. In the saliva [60] and gut mucins [61] of mammals, GlcN is a central chemical constituent such as GalN, which might be also true for bacterial and fungal EPS. However, it remains uncertain whether the molar 1:1 ratio of GlcN: and GalN repeatedly stated as textbook knowledge is really true for the murein of all bacterial species.

### Interactions among EPS constituents

In this study, most of the variation in EPS composition happened due to microbial type and was seen by different amounts of proteins, amino sugars, and DNA. This might indicate shifts in EPS composition are linked to ‘microbial type’, or intrinsic microbial characteristics and taxonomy. Contrastingly, EPS-carbohydrate production was mainly affected by extracellular environmental conditions. For this reason, changes in EPS quantities will also likely reflect fluctuations in environmental conditions, as carbohydrates as biofilms are mostly made up of them [6]. In our study, this trend was especially seen in bacterial cultures, however, fungal EPS production and composition was mainly driven by the presence of the quartz matrix and the complexity of the carbon source and increases in fungal EPS production were not only reflected by their carbohydrate but also by their GalN and ManN concentrations. This finding suggests that fungal EPS production is more affected by extracellular environmental factors than bacterial EPS production, even though the reasons behind these differences still remain unclear.

## Conclusions

Changes in microbial EPS quantities were driven in the current study by external conditions, namely substrate quality and surface presence. These changes were reflected by the amounts of EPS-carbohydrates, whereas shifts in EPS composition were mostly caused by microbial characteristics, i.e., microbial type, species-specific cell wall composition, etc. This is particularly true for bacterial species. In contrast, fungal EPS production and composition generally showed a stronger response to substrate quality and surface presence and less effects of species properties. Particularly EPS-GlcN represented the fungal reaction to the quartz matrix, whereas ManN, GalN, and carbohydrate concentrations were more related to increases in fungal EPS production, particularly in the presence of the more complex starch. EPS-MurN and EPS-DNA generally indicated the presence of bacterial necromass, which might be also true for EPS-GlcN to an unknown extent for bacterial and fungal species. The EPS-carbohydrate/protein ratio increased with substrate complexity and seems to be a powerful indicator of EPS functioning. An increased EPS-carbohydrate/protein ratio might be related to higher hydrophobicity, representing a stronger tolerance against water, solvents, and biocides. However, this view needs more experimental evidence. The current study revealed that the CER extraction method is a powerful tool to investigate the EPS production of cultured bacteria and fungi. This could be an excellent approach for further detailed investigations of the EPS composition in soils and sediments.

## Supplementary Information

Supplementary Material 1.

Supplementary Material 2.

Supplementary Material 3.

Supplementary Material 4.

## Data Availability

The datasets generated during the current study are available from the corresponding author upon reasonable request.
