# Supplementary material for: Constituent of extracellular polymeric substances (EPS) produced by a range of soil bacteria and fungi
Source: BMC Microbiol. 2025 May 15;25:298. doi: 10.1186/s12866-025-04034-z (PMC12079940; doi:10.1186/s12866-025-04034-z)
Supplement: Supplementary file 1 — Supplementary Material 1. [file 12866_2025_4034_MOESM1_ESM.pptx]

## Slide 1
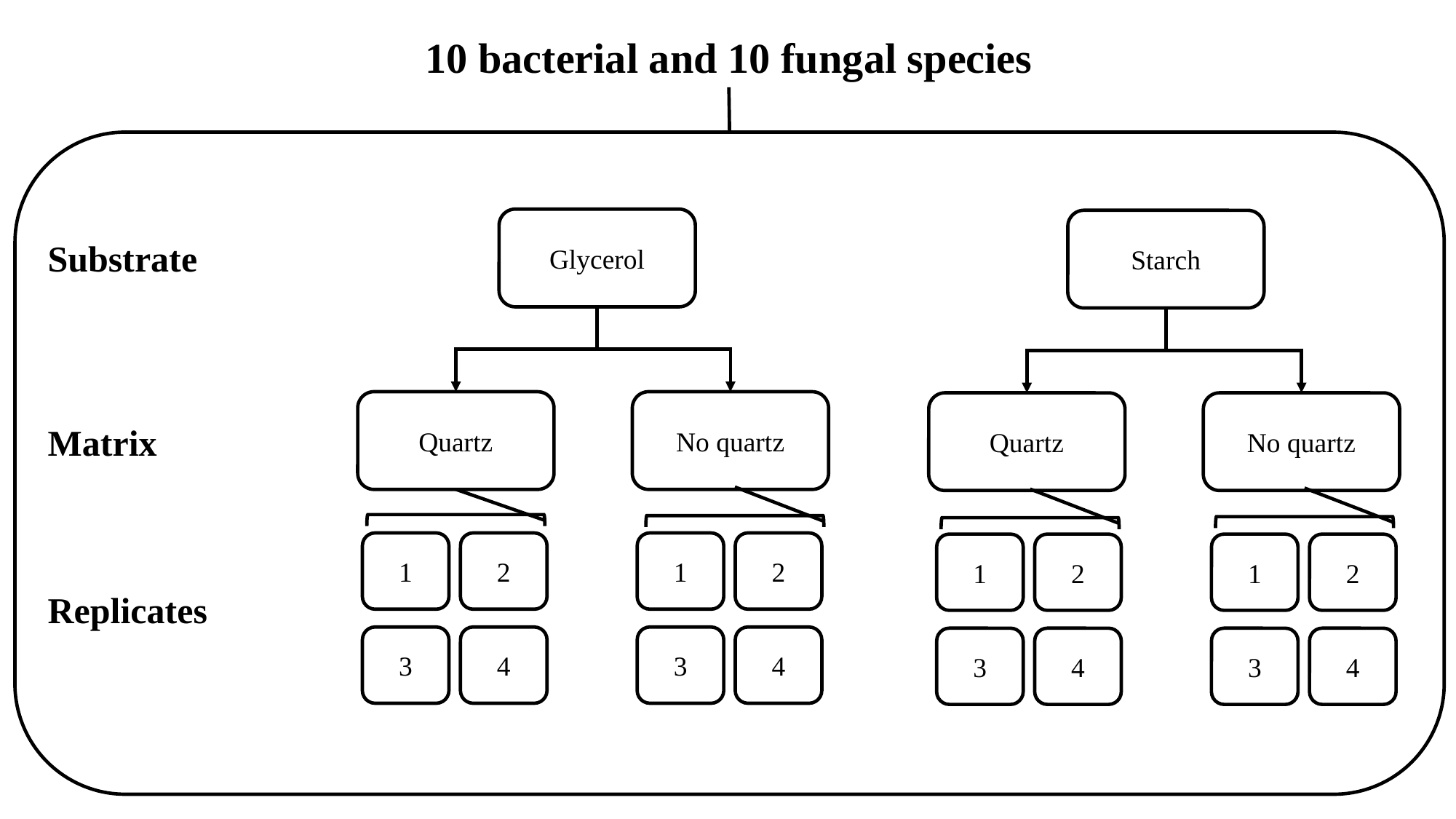

10 bacterial and 10 fungal species
Glycerol
Starch
Substrate
Quartz
No quartz
Quartz
No quartz
Matrix
1
2
1
2
1
2
1
2
Replicates
4
4
3
3
4
4
3
3
