# Supplementary material for: Constituent of extracellular polymeric substances (EPS) produced by a range of soil bacteria and fungi
Source: BMC Microbiol. 2025 May 15;25:298. doi: 10.1186/s12866-025-04034-z (PMC12079940; doi:10.1186/s12866-025-04034-z)
Supplement: Supplementary file 2 — Supplementary Material 2. [file 12866_2025_4034_MOESM2_ESM.docx]

**Shifts in the composition of extracellular polymeric substances (EPS) produced by a range of soil bacteria and fungi**

Oliva R.L.^1^, Khadka U.B.^1^, Camenzind T.^2^, Dyckmans J.^3^, Jörgensen R.G.^1^

^1^ Department of Soil Biology and Plant Nutrition, University of Kassel, Nordbahnhofstr. 1a, D-37213 Witzenhausen, Germany

^2^ Institute of Biology, Freie Universität Berlin, Altensteinstr. 6, 14195 Berlin, Germany

^3^ Institute of Soil Science and Forest Nutrition, University of Göttingen, Büsgenweg 2, 37077 Göttingen, Germany

* Corresponding author: [rebeca.oliva@uni-kassel.de](mailto:rebeca.oliva@uni-kassel.de)

Soil Biology and Plant Nutrition, University of Kassel

Nordbahnhofstr. 1a, 37213 Witzenhausen, Germany

**Supplemental Information 1 (S1)**

Table S1. Detailed information on all the species used for this study and their origin.

| **Species** | **Source** | **Phylum** | **Gram (+)/(-)** |
| --- | --- | --- | --- |
| Bacteria | | | |
| *Agrobacterium radiobacter* | DSMZ - DSM 13874 | Proteobacteria | (-) |
| *Arthrobacter globiformis* | DSMZ - DSM 20124 | Actinobacteria | (+) |
| *Bacillus subtilis* | DSMZ - DSM 10 | Firmicutes | (+) |
| *Escherichia coli* | FU Berlin - MG1655 | Proteobacteria | (-) |
| *Micrococcus luteus* | DSMZ - DSM 20030 | Actinobacteria | (+) |
| *Micromonospora rosaria* | DSMZ - DSM 803 | Actinobacteria | (+) |
| *Mycobacterium phlei* | DSMZ - DSM 43214 | Actinobacteria | (+) |
| *Nocardioides soli* | DSMZ - DSM 105498 | Actinobacteria | (+) |
| *Pseudomonas fluorescens* | DSMZ - DSM 50090 | Proteobacteria | (-) |
| *Streptomyces griseus* | DSMZ - DSM 40236 | Actinobacteria | (+) |
| Fungi | | | |
| *Arcopilus cupreus* | FU Berlin | Ascomycota | NA |
| *Bionectria solani* | FU Berlin | Ascomycota | NA |
| *Cladosporium delicatulum* | FU Berlin | Ascomycota | NA |
| *Epicoccum dendrobii* | FU Berlin | Ascomycota | NA |
| *Fusarium acutatum* | FU Berlin | Ascomycota | NA |
| *Mortierella gemmifera* | FU Berlin | Mucoromycota | NA |
| *Mucor moelleri* | FU Berlin | Mucoromycota | NA |
| *Penicillium javanicum* | FU Berlin | Ascomycota | NA |
| *Trichoderma hamatum* | FU Berlin | Ascomycota | NA |
| *Umbelopsis vinaceae* | FU Berlin | Mucoromycota | NA |
