# Supplementary material for: Constituent of extracellular polymeric substances (EPS) produced by a range of soil bacteria and fungi
Source: BMC Microbiol. 2025 May 15;25:298. doi: 10.1186/s12866-025-04034-z (PMC12079940; doi:10.1186/s12866-025-04034-z)
Supplement: Supplementary file 3 — Supplementary Material 3. [file 12866_2025_4034_MOESM3_ESM.docx]

**Constituent of extracellular polymeric substances (EPS) produced by a range of soil bacteria and fungi**

Oliva R.L.^1^, Khadka U.B.^1^, Camenzind T.^2^, Dyckmans J.^3^, Jörgensen R.G.^1^

^1^ Department of Soil Biology and Plant Nutrition, University of Kassel, Nordbahnhofstr. 1a, D-37213 Witzenhausen, Germany

^2^ Institute of Biology, Freie Universität Berlin, Altensteinstr. 6, 14195 Berlin, Germany

^3^ Institute of Soil Science and Forest Nutrition, University of Göttingen, Büsgenweg 2, 37077 Göttingen, Germany

* Corresponding author: [rebeca.oliva@uni-kassel.de](mailto:rebeca.oliva@uni-kassel.de)

Soil Biology and Plant Nutrition, University of Kassel

Nordbahnhofstr. 1a, 37213 Witzenhausen, Germany

**Supporting Information S2**

Fungal isolation was carried out in three steps: (1) soil washing to remove spores in order to capture active hyphae only, (2) fungal isolation on different carbon substrates and (3) culturing and identification of fungal isolates. In the first step, soils were sieved to 2mm and shaken with a 0.1% sodium pyrophosphate solution for one hour. Thereafter, soil was transferred on a 53 µm mesh and washed thoroughly (1). For fungal isolation, different dilutions of remaining soil particles were plated on petri dishes containing certain carbon sources - starch in case of isolates used in this study - supplemented with a nutrient solution. After 3-6 days of growth individual colonies were picked and transferred first to a medium based on the same carbon source, using a defined phytagel-based medium (2), and in a second step transferred to malt-extract agar for further culturing. Fungal identity was determined by Sanger-sequencing, generating long sequences of SSU, ITS and LSU regions with the primers ITS1 and LR3. Individual marker regions were compared to respective databases to determine fungal identity – ITS to Unite database (3) and LSU to RDP LSU dataset (4). Comparisons of these isolate sequence identities with fungal community data from the same soil samples allowed to select for fungal isolates abundant in these soils (based on Illumina sequencing data).

**References for Supplementary Material S2**

1. Parkinson D, Williams ST. A method for isolating fungi from soil microhabitats. Plant and soil. 1960; 13, 347-355
2. Leifheit EF, Camenzind T, Lehmann A, Andrade-Linares DR, Fussan M, Westhusen S, Rillig MC. Fungal traits help to understand the decomposition of simple and complex plant litter. bioRxiv. 2023; 2022-12
3. Nilsson RH, Larsson KH, Taylor AFS, Bengtsson-Palme J, Jeppesen TS, Schigel D, Abarenkov K. The UNITE database for molecular identification of fungi: handling dark taxa and parallel taxonomic classifications. Nucleic acids research. 2018; 47(D1), D259-D264
4. Cole JR, Wang Q, Fish JA, Chai B, McGarrell DM, Sun Y, Tiedje JM. Ribosomal Database Project: data and tools for high throughput rRNA analysis. Nucleic acids research. 2014; 42(D1), D633-D642
