# Supplementary material for: Constituent of extracellular polymeric substances (EPS) produced by a range of soil bacteria and fungi
Source: BMC Microbiol. 2025 May 15;25:298. doi: 10.1186/s12866-025-04034-z (PMC12079940; doi:10.1186/s12866-025-04034-z)
Supplement: Supplementary file 4 — Supplementary Material 4. [file 12866_2025_4034_MOESM4_ESM.docx]

**Constituent of extracellular polymeric substances (EPS) produced by a range of soil bacteria and fungi**

Oliva R.L.^1^, Khadka U.B.^1^, Camenzind T.^2^, Dyckmans J.^3^, Jörgensen R.G.^1^

^1^ Department of Soil Biology and Plant Nutrition, University of Kassel, Nordbahnhofstr. 1a, D-37213 Witzenhausen, Germany

^2^ Institute of Biology, Freie Universität Berlin, Altensteinstr. 6, 14195 Berlin, Germany

^3^ Institute of Soil Science and Forest Nutrition, University of Göttingen, Büsgenweg 2, 37077 Göttingen, Germany

* Corresponding author: [rebeca.oliva@uni-kassel.de](mailto:rebeca.oliva@uni-kassel.de)

Soil Biology and Plant Nutrition, University of Kassel

Nordbahnhofstr. 1a, 37213 Witzenhausen, Germany

**Supporting Information S3**

Table. S3 Amounts quantified in extracted EPS of twenty microbial species grown under different substrate and matrix treatments. Results are expressed in µg ml^-1^ of cell culture (except DNA, expressed in ng ml^-1^ of cell culture) after 4-day incubation and CV represents the coefficient of variation between replicates (n=4).

| Microbial Type | Species | Treatment | Proteins | Carbohydrates | MurN | ManN | GalN | GlcN | DNAa |
| --- | --- | --- | --- | --- | --- | --- | --- | --- | --- |
|  |  |  | µg ml^-1^ of cell culture | | | | | | ng ml^-1^ of cell culture |
| Bacteria | *B.subtilis* | Quartz+glycerol | 1383 | 1011 | 60.93 | 16.74 | 4.95 | 68.38 | 0.36 |
|  |  | Glycerol | 188 | 107 | 4.84 | 10.22 | 5.62 | 9.51 | 0.32 |
|  |  | Quartz+starch | 131 | 61293 | 23.62 | 11.23 | 3.73 | 27.17 | 0.22 |
|  |  | Starch | 230 | 1336 | 4.29 | 10.28 | 3.64 | 14.95 | 0.24 |
|  | *M.phlei* | Quartz+glycerol | 427 | 1108 | 32.15 | 15.24 | 9.30 | 45.14 | 0.31 |
|  |  | Glycerol | 74 | 102 | 6.33 | 11.00 | 6.62 | 9.35 | 0.31 |
|  |  | Quartz+starch | 212 | 86087 | 25.46 | 11.24 | 4.58 | 27.60 | 0.21 |
|  |  | Starch | 306 | 2021 | 5.07 | 10.99 | 5.91 | 14.64 | 0.31 |
|  | *M.luteus* | Quartz+glycerol | 210 | 45 | 3.27 | 11.99 | 7.23 | 16.38 | 0.35 |
|  |  | Glycerol | 403 | 80 | 1.62 | 12.41 | 8.80 | 26.30 | 0.35 |
|  |  | Quartz+starch | 50 | 70654 | 1.10 | 11.37 | 5.67 | 9.00 | 0.27 |
|  |  | Starch | 64 | 291 | 0.75 | 10.08 | 5.06 | 10.04 | 0.33 |
|  | *P.fluorescens* | Quartz+glycerol | 259 | 1512 | 2.40 | 11.48 | 5.28 | 12.74 | 0.36 |
|  |  | Glycerol | 720 | 91 | 3.19 | 21.14 | 9.85 | 31.12 | 0.33 |
|  |  | Quartz+starch | 62 | 72159 | 0.67 | 9.90 | 4.56 | 7.89 | 0.16 |
|  |  | Starch | 50 | 241 | 0.74 | 9.84 | 5.04 | 9.61 | 0.28 |
|  | *A.radiobacter* | Quartz+glycerol | 328 | 236 | 2.78 | 8.95 | 3.47 | 6.13 | 0.29 |
|  |  | Glycerol | 30 | 55 | 0.38 | 8.67 | 3.31 | 4.28 | 0.24 |
|  |  | Quartz+starch | 101 | 67437 | 0.07 | 8.96 | 3.33 | 4.63 | 0.06 |
|  |  | Starch | 27 | 1599 | 0.49 | 8.69 | 3.29 | 4.12 | 0.12 |
|  | *S.griseus* | Quartz+glycerol | 429 | 148 | 15.68 | 10.29 | 5.32 | 14.07 | 0.24 |
|  |  | Glycerol | 64 | 4 | 2.78 | 9.70 | 3.17 | 4.28 | 0.00 |
|  |  | Quartz+starch | 87 | 71709 | 3.52 | 9.68 | 3.36 | 7.99 | 0.24 |
|  |  | Starch | 59 | 2578 | 0.24 | 9.74 | 3.18 | 4.66 | 0.15 |
|  | *A.globiformis* | Quartz+glycerol | 920 | 533 | 13.04 | 75.02 | 3.93 | 43.58 | 0.38 |
|  |  | Glycerol | 49 | 49 | 1.88 | 12.15 | 3.33 | 6.97 | 0.14 |
|  |  | Quartz+starch | 82 | 59704 | 10.26 | 10.34 | 2.58 | 10.21 | 0.26 |
|  |  | Starch | 78 | 855 | 1.05 | 9.79 | 2.32 | 5.06 | 0.24 |
|  | *N.soli* | Quartz+glycerol | 440 | 84 | 0.12 | 9.88 | 2.45 | 4.68 | 0.29 |
|  |  | Glycerol | 39 | 4 | 0.09 | 9.73 | 2.30 | 4.01 | 0.05 |
|  |  | Quartz+starch | 154 | 64470 | 0.09 | 9.85 | 2.41 | 6.48 | 0.24 |
|  |  | Starch | 41 | 1691 | 0.11 | 7.85 | 4.66 | 7.73 | 0.18 |
|  | *E.coli* | Quartz+glycerol | 399 | 253 | 5.39 | 31.29 | 3.26 | 18.31 | 0.30 |
|  |  | Glycerol | 90 | 76 | 0.79 | 10.18 | 2.98 | 5.09 | 0.21 |
|  |  | Quartz+starch | 95 | 105752 | 3.83 | 9.71 | 2.77 | 7.11 | 0.19 |
|  |  | Starch | 21 | 1362 | 1.71 | 8.78 | 3.42 | 5.64 | 0.24 |
|  | *M.rosaria* | Quartz+glycerol | 302 | 189 | 0.42 | 8.43 | 5.22 | 6.71 | 0.27 |
|  |  | Glycerol | 13 | 40 | 0.23 | 8.05 | 5.07 | 5.32 | 0.11 |
|  |  | Quartz+starch | 102 | 84911 | 0.28 | 8.51 | 5.49 | 7.82 | 0.23 |
|  |  | Starch | 15 | 4088 | 0.24 | 8.07 | 5.11 | 5.57 | 0.20 |
| Fungi | *M.moelleri* | Quartz+glycerol | 400 | 321 | ----- | 2.47 | 1.87 | 2.73 | 0.18 |
|  |  | Glycerol | 15 | 301 | ----- | 2.41 | 1.44 | 1.32 | 0.00 |
|  |  | Quartz+starch | 57 | 64936 | ----- | 2.57 | 2.04 | 8.93 | 0.03 |
|  |  | Starch | 17 | 3294 | ----- | 2.41 | 1.43 | 2.16 | 0.01 |
|  | *F.acutatum* | Quartz+glycerol | 436 | 221 | ----- | 2.57 | 1.74 | 11.34 | 0.15 |
|  |  | Glycerol | 21 | 314 | ----- | 5.31 | 1.79 | 2.71 | 0.01 |
|  |  | Quartz+starch | 66 | 66068 | ----- | 7.51 | 2.49 | 10.60 | 0.04 |
|  |  | Starch | 39 | 16856 | ----- | 6.62 | 2.51 | 10.88 | 0.06 |
|  | *M.gemmifera* | Quartz+glycerol | 526 | 143 | ----- | 6.57 | 2.50 | 4.27 | 0.00 |
|  |  | Glycerol | 12 | 247 | ----- | 6.06 | 2.47 | 2.50 | 0.00 |
|  |  | Quartz+starch | 129 | 61729 | ----- | 6.71 | 2.52 | 6.01 | 0.00 |
|  |  | Starch | 14 | 2028 | ----- | 5.53 | 2.78 | 2.71 | 0.00 |
|  | *A.cupreus* | Quartz+glycerol | 514 | 147 | ----- | 5.14 | 3.44 | 5.63 | 0.08 |
|  |  | Glycerol | 19 | 244 | ----- | 4.88 | 3.10 | 2.88 | 0.00 |
|  |  | Quartz+starch | 50 | 61907 | ----- | 5.33 | 3.23 | 6.22 | 0.01 |
|  |  | Starch | 32 | 12090 | ----- | 5.12 | 3.16 | 4.93 | 0.01 |
|  | *U.vinacea* | Quartz+glycerol | 498 | 251 | ----- | 5.16 | 4.38 | 4.76 | 0.13 |
|  |  | Glycerol | 54 | 32 | ----- | 4.17 | 1.83 | 2.28 | 0.06 |
|  |  | Quartz+starch | 149 | 65932 | ----- | 3.30 | 1.89 | 7.56 | 0.00 |
|  |  | Starch | 24 | 2192 | ----- | 3.32 | 0.65 | 1.87 | 0.02 |
|  | *C.delicatum* | Quartz+glycerol | 457 | 107 | ----- | 3.44 | 0.79 | 2.71 | 0.11 |
|  |  | Glycerol | 35 | 311 | ----- | 3.21 | 0.45 | 0.74 | 0.01 |
|  |  | Quartz+starch | 73 | 66029 | ----- | 3.58 | 0.94 | 3.76 | 0.04 |
|  |  | Starch | 24 | 2117 | ----- | 5.13 | 1.53 | 2.10 | 0.03 |
|  | *T.hammatum* | Quartz+glycerol | 353 | 233 | ----- | 7.14 | 2.39 | 7.16 | 0.07 |
|  |  | Glycerol | 144 | 148 | ----- | 7.03 | 2.11 | 4.95 | 0.00 |
|  |  | Quartz+starch | 84 | 60968 | ----- | 7.37 | 2.13 | 7.15 | 0.03 |
|  |  | Starch | 61 | 25139 | ----- | 7.07 | 2.13 | 6.67 | 0.00 |
|  | *B.solani* | Quartz+glycerol | 408 | 190 | ----- | 7.22 | 2.20 | 4.58 | 0.04 |
|  |  | Glycerol | 126 | 80 | ----- | 6.90 | 2.39 | 4.03 | 0.00 |
|  |  | Quartz+starch | 116 | 46760 | ----- | 6.73 | 4.75 | 6.45 | 0.00 |
|  |  | Starch | 22 | 1218 | ----- | 6.69 | 2.74 | 3.91 | 0.01 |
|  | *E.dendrobii* | Quartz+glycerol | 324 | 137 | ----- | 6.72 | 2.87 | 5.96 | 0.01 |
|  |  | Glycerol | 26 | 45 | ----- | 6.75 | 2.69 | 3.94 | 0.00 |
|  |  | Quartz+starch | 102 | 19851 | ----- | 8.98 | 3.06 | 8.21 | 0.05 |
|  |  | Starch | 166 | 778 | ----- | 8.82 | 3.38 | 11.58 | 0.07 |
|  | *P.javanicum* | Quartz+glycerol | 467 | 234 | ----- | 6.09 | 2.18 | 12.50 | 0.16 |
|  |  | Glycerol | 51 | 81 | ----- | 5.57 | 1.88 | 3.27 | 0.02 |
|  |  | Quartz+starch | 134 | 35399 | ----- | 6.27 | 2.37 | 8.21 | 0.04 |
|  |  | Starch | 21 | 1558 | ----- | 5.62 | 2.15 | 3.06 | 0.00 |
| CV (±%) |  |  | 22 | 14 | 14 | 4 | 7 | 12 | 32 |
